# A phase II experience with neoadjuvant irinotecan (CPT-11), 5-fluorouracil (5-FU) and leucovorin (LV) for colorectal liver metastases

**DOI:** 10.1186/1471-2407-9-156

**Published:** 2009-05-20

**Authors:** Oliver F Bathe, Scott Ernst, Francis R Sutherland, Elijah Dixon, Charles Butts, David Bigam, David Holland, Geoffrey A Porter, Jennifer Koppel, Scot Dowden

**Affiliations:** 1 Department of Surgery, University of Calgary, Calgary, Canada; 2 Department of Oncology, University of Calgary, Calgary, Canada; 3 Department of Oncology, London Regional Cancer Centre, London, Canada; 4 Department of Oncology, University of Alberta, Edmonton, Canada; 5 Department of Surgery, University of Alberta, Edmonton, Canada; 6 Department of Oncology, Lethbridge Cancer Centre, Lethbridge, Canada; 7 Department of Surgery, Dalhousie University, Halifax, Canada

## Abstract

**Background:**

Chemotherapy may improve survival in patients undergoing resection of colorectal liver metastases (CLM). Neoadjuvant chemotherapy may help identify patients with occult extrahepatic disease (averting unnecessary metastasectomy), and it provides *in vivo *chemosensitivity data.

**Methods:**

A phase II trial was initiated in which patients with resectable CLM received CPT-11, 5-FU and LV for 12 weeks. Metastasectomy was performed unless extrahepatic disease appeared. Postoperatively, patients with stable or responsive disease received the same regimen for 12 weeks. Patients with progressive disease received either second-line chemotherapy or best supportive care. The primary endpoint was disease-free survival (DFS); secondary endpoints included overall survival (OS) and safety.

**Results:**

35 patients were accrued. During preoperative chemotherapy, 16 patients (46%) had grade 3/4 toxicities. Resection was not possible in 5 patients. One patient died of arrhythmia following surgery, and 1 patient had transient liver failure. During the postoperative treatment phase, 12 patients (55%) had grade 3/4 toxicities. Deep venous thrombosis (DVT) occurred in 11 patients (34%) at various times during treatment. Of those who underwent resection, median DFS was 23.0 mo. and median OS has not been reached. The overall survival from time of diagnosis of liver metastases was 51.6 mo for the entire cohort.

**Conclusion:**

A short course of chemotherapy prior to hepatic metastasectomy may serve to select candidates best suited for resection and it may also direct postoperative systemic treatment. Given the significant incidence of DVT, alternative systemic neoadjuvant regimens should be investigated, particularly those that avoid the use of a central venous line.

**Trial Registration:**

ClinicalTrials.gov NCT00168155.

## Background

The liver is the most common site of metastasis for colorectal cancer. Resection is the only hope for long-term survival for CLM. The median survival in such patients prior to recent advances in chemotherapy ranged from 19 – 30 mo, with five year survivals of 30–39% [1-3]. Recent studies have reported even better survivals [1,2,4,5]. This may be due to improved chemotherapy, administered during various times of the patient's illness. Typically, response rates for first line chemotherapy are 39–60% [6-9]. While improvements in survival have been reported in unresectable metastatic colorectal cancer, there is little evidence on how best to administer these agents in the setting of resectable CLM.

We have recently published a detailed description of a phase II clinical trial involving the perioperative administration of CPT-11, 5-FU and LV to patients with resectable CLM [10]. Neoadjuvant chemotherapy has a number of potential advantages in this setting. First, the degree of response provides information on the *in vivo *chemosensitivity of tumors. This may help to determine the appropriateness of administering the same chemotherapy after resection. In non-responders, the toxicities of these agents are avoided and alternatives can be considered. Administration of preoperative chemotherapy may also enable selection of candidates for resection. In particular, patients who develop extrahepatic disease during a short course of chemotherapy were likely unsuitable candidates for resection in the first place. Finally, in some instances, neoadjuvant chemotherapy may enhance resectability, as reductions in tumor volume may limit the amount of the liver that will need to be removed to eradicate all gross disease [11]. We herein report our experience with this approach. Importantly, we describe our results in an intent-to-treat fashion.

## Methods

The trial was registered with ClinicalTrials.gov (registration #NCT00168155). The trial was approved by the Conjoint Health Research Ethics Board at the University of Calgary, as well as by the local Ethics committees of collaborating participating institutions. Informed, written consent was obtained from all patients. A detailed description of the protocol was recently reported [10]. In brief, the general aim was to determine the efficacy of neoadjuvant chemotherapy for patients with resectable CLM in reducing recurrence rate. The primary objective was to evaluate disease-free survival. Secondary objectives included documentation of safety and evaluation of overall survival.

### Inclusion and Exclusion Criteria

Inclusion criteria included the following: (a) patients with Stage IV colorectal adenocarcinoma isolated to the liver; (b) at least one radiographically measurable lesion; (c) determination by a hepatobiliary surgeon that the liver metastases were amenable to complete resection and/or radiofrequency ablation (RFA). Exclusion criteria included the following: (a) the primary tumor was not controlled by locoregional treatments; and (b) previous chemotherapy for metastatic colorectal cancer.

A biopsy was initially required. However, in the latter stages of the trial, enrollment was also permitted in patients with all of the following criteria: liver lesions with typical features of CLM on MRI and CT; FDG uptake on PET imaging; and elevated serum CEA. These latter criteria only applied to two patients.

### Treatments

Chemotherapy was administered for 12 weeks in the preoperative period. At 6 – 8 weeks, the clinical response was assessed by abdominal CT. If progression was not identified, the remaining cycles of chemotherapy were given. The first two patients received chemotherapy as described by Saltz et al. [6]. Chemotherapy was administered as per Douillard and colleagues [7] in 30 patients, and the remaining patients received chemotherapy as per the FOLFIRI regimen [12]. The reason for changes in regimen were related to changes in standards of practice for palliative treatment of metastatic colorectal cancer during the trial. The method of vascular access for administration of chemotherapy was at the discretion of the medical oncologist.

The extent of hepatic resection was determined by the surgeon. A resection margin of at least 1 cm was the goal. In patients where one or more of the CLM was not felt to be resectable for either technical or patient-related reasons, RFA was performed with the goal of a 1 cm ablation margin. All RFAs were done in an open fashion.

All patients with stable and responsive disease following preoperative chemotherapy who had undergone liver resection were offered 12 additional cycles of the same chemotherapy. The goal was to initiate adjuvant chemotherapy 21 – 72 days after surgery. Patients with progressive disease were offered an alternative agent or best supportive care.

Prophylactic anticoagulants were not administered routinely during chemotherapy. Standard antithrombotic prophylaxis prior to surgery consisted of administration of subcutaneous heparin and application of intermittent compression stockings.

### Data Analysis

A detailed analysis plan and definitions related to outcomes were previously published [10]. Response to chemotherapy was determined by RECIST criteria [13]. Date of diagnosis was considered the date of biopsy-proven CLM, except in the case of synchronous CLM (in which case the date of diagnosis was considered the date of surgery for the primary tumor) or in cases where diagnosis was clinical (in which case the date of the final qualifying diagnostic test was considered the date of diagnosis).

It was estimated that 70 patients would be required to demonstrate an improvement in 2-year disease-free survival of 25%, given an estimated historical 3 year disease-free survival of 40% (i.e, P_0_), with an alpha 0.05 and a power of 0.8, by 2-tailed test. The study was stopped prematurely due to safety concerns as outlined below.

Survival was estimated by the method of Kaplan-Meier. The log-rank test was used to test for differences in survival times. A p-value of < 0.05 was considered significant. All analyses were made on an intent-to-treat basis, since that would provide the most meaningful information for a cohort of patients considered upfront to have resectable disease.

Adverse events related to chemotherapy were graded according to CTCAE Version 3.0 http://ctep.info.nih.gov. Adverse events were attributed to the chemotherapy if they occurred within 30 days of the last dose. Operative mortality was defined as death within 30 days of operation or during the same hospitalization.

## Results

### Patients

From December, 2001 – July, 2005, 35 patients were accrued. The characteristics of this cohort are summarized in Table 1. Synchronous CLM were defined as the discovery of CLM ≤ 2 months after diagnosis of colorectal cancer. Of those with metachronous tumors (40%), the median disease-free interval was 24 mo (range 4 – 61); adjuvant chemotherapy had been administered prior to accrual in 11 of those patients. The average number of CLM was 2 ± 3 (range: 1 – 20). The largest lesions measured 5.0 ± 2.6 cm (range: 1.4 – 13.7 cm).

**Table 1:** Patient characteristics at time of accrual.

Parameter	
**Total Number of Patients**	35
**Age**	59 ± 8 years
**Gender**	
Male	24 (69%)
Female	11 (31%)
**Site of Primary Tumor**	
Colon	22 (63%)
Rectosigmoid	7 (20%)
Rectum	6 (17%)
**Previous Treatment for Primary Tumor**	
Chemotherapy	11 (31%)
Radiotherapy	6 (17%)
**Initial Staging**	
Stage I	1 (3%)
Stage II	4 (11%)
Stage III	9 (26%)
Stage IV (ie: synchronous liver metastases)	21 (60%)
**Metachronous liver metastases**	14 (40%)
Median disease-free interval (range)	24 mo (4–61 mo)
**Mean Number of Liver Lesions**	2 ± 3
Solitary metastasis	21 (60%)
> 1 metastases	14 (40%)
**Size of Largest Liver Lesion**	5.0 ± 2.6 cm
≤ 5 cm	21 (60%)
> 5 cm	14 (40%)
**Carcinoembryogenic Antigen**	31 ± 41 ng/mL

### Treatment Pathways

**Figure 1:**
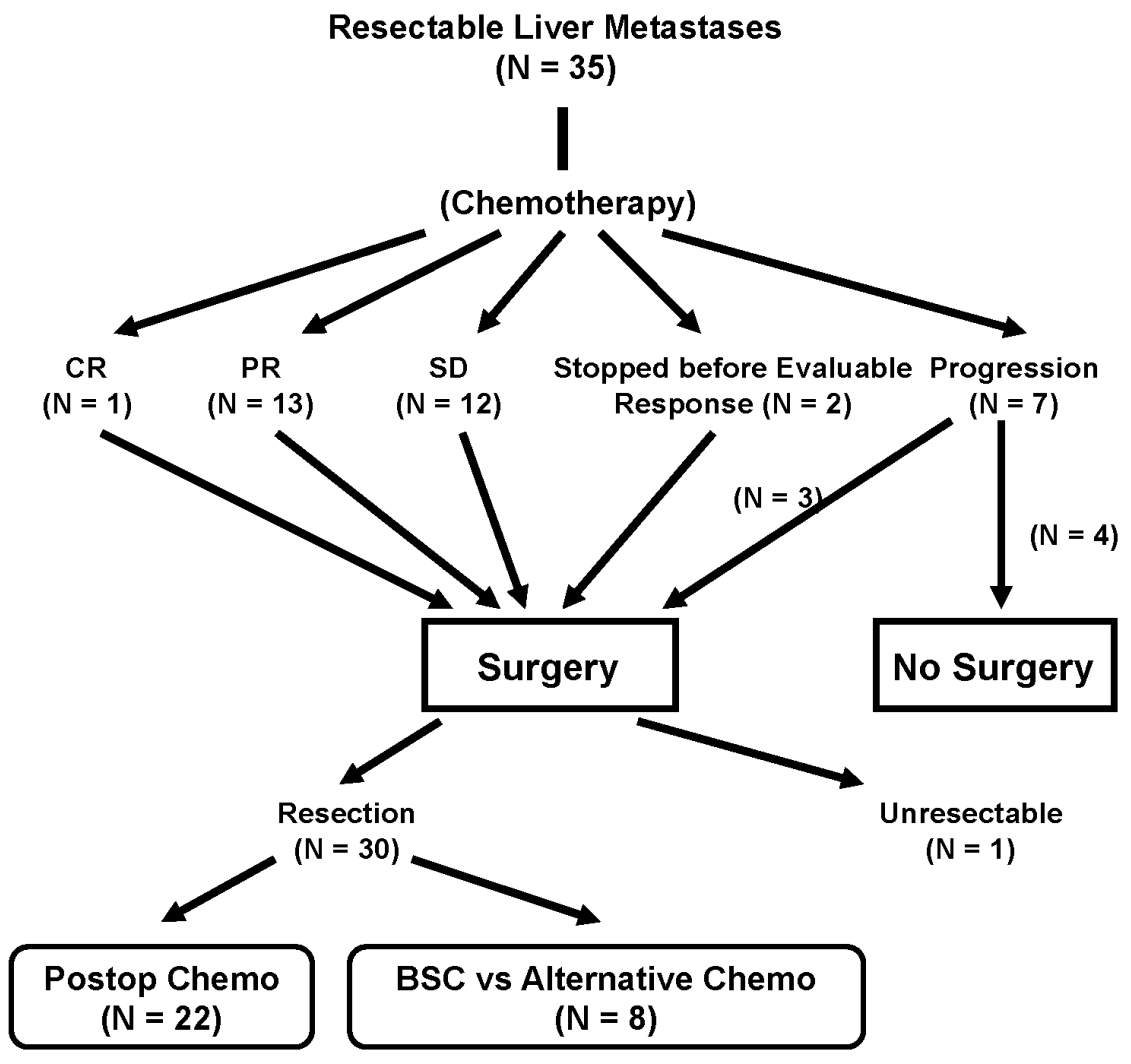
**Distribution of patients who received each phase of treatment**.

Treatments administered to the study cohort are summarized in Figure 1. Two patients stopped their preoperative chemotherapy before their response was evaluated (one due to refusal related to anxiety from the delay of surgery; the other because of the appearance of a DVT, which prompted that individual to decline further chemotherapy). A response to chemotherapy was seen in 14 patients (40%; 1 CR and 13 PR); 12 patients (36%) had stable disease; and 7 patients (18%) had progression. All radiographic responses were confirmed pathologically. Two of the patients with progressive disease were identified during the mid-interval CT; increased tumor size but no new metastases were observed. Surgery was ultimately performed on 31 patients, of whom 30 (97%) underwent successful R0 resection or ablation. One patient was found at laparotomy to have extrahepatic disease. Laparotomy was not performed in 4 patients, due to the appearance of extrahepatic disease (N = 2), clinical deterioration (N = 1), or refusal (N = 1). Twenty-two patients received postoperative chemotherapy. These comprised patients with measurable responses or stable disease. Four patients with stable disease did not receive postoperative chemotherapy because of refusal (N = 2); poor postoperative recovery (N = 1); or surgical death (N = 1). Patients with progression who underwent resection did not receive postoperative chemotherapy. Patients who progressed to nonresectability were all given second-line chemotherapy (FOLFOX).

Average time from last dose of preoperative chemotherapy to surgery was 50 ± 34 d. Average time from the date of surgery to the initiation of postoperative chemotherapy was 58 ± 11 d.

### Treatment-related Toxicities

The toxicities related to preoperative chemotherapy are outlined in Table 2. Seven patients (20%) did not receive all scheduled courses of chemotherapy and two of those were because of disease progression seen on interval CT. Severe adverse events occurred in 6 patients (14%), including DVT/PE (N = 1), myocardial infarction (N = 1), pseudomembranous colitis (N = 1), pneumonia (N = 1), small bowel perforation (N = 1), and hypokalemia (N = 1).

**Table 2:** Adverse events based on worst grade toxicity experienced during preoperative and postoperative chemotherapy.

Adverse Event	Preoperative Chemotherapy (n = 35)		Postoperative Chemotherapy (n = 22)	
	
	Grade 1/2 N (%)	Grade 3/4 N (%)	Grade 1/2 N (%)	Grade 3/4 N (%)
**Gastrointestinal**				
Nausea/vomiting	25 [71]	0 (0)	17 (77)	0 (0)
Diarrhea	19 (54)	2 (6)	11 (50)	1 (5)
Constipation	8 (23)	1 (3)	8 (36)	0 (0)
Anorexia	7 (20)	0 (0)	3 (14)	0 (0)
Mucositis/stomatitis	11 (31)	0 (0)	8 (36)	0 (0)
Small Bowel Perforation	0 (0)	1 (3)	0 (0)	0 (0)
Other: heartburn, dyspepsia, gas pain, taste alteration, dysgeusia, hiccups, dry mouth, abdominal cramping, dysphagia	12 (34)	0 (0)	11 (50)	0 (0)

**Constitutional**				
Fatigue or lethargy or insomnia	30 (86)	0 (0)	16 (73)	0 (0)
Diaphoresis/flushing	4 (11)	0 (0)	1 (5)	0 (0)
Other: fever, cough, cold	3 (9)	0 (0)	1 (5)	0 (0)

**Infection**	10 (29)	2 (6)	5 (23)	2 (9)

**Pain**	7 (20)	0 (0)	7 (32)	0 (0)

**Laboratory: Hematological**				
Lymphopenia	1 (3)	2 (6)	2 (9)	0 (0)
Neutropenia	12 (34)	4 (11)	8 (36)	9 (41)
Anemia	13 (37)	0 (0)	9 (41)	0 (0)
Thrombocytopenia	4 (11)	0 (0)	8 (36)	0 (0)

**Laboratory: Biochemistry**				
Elevated liver function tests: AST, ALT, bilirubin, alkaline phosphatase, LDH	6 (17)	0 (0)	5 (23)	0 (0)
Elevated creatinine	2 (6)	0 (0)	1 (5)	0 (0)
Decreased protein/albumin	2 (6)	0 (0)	2 (9)	0 (0)
Coagulation abnormalities: INR/PTT	0 (0)	1 (3)	0 (0)	1 (5)
Miscellaneous electrolyte and metabolic disturbances: hypermagnesemia, elevated phosphorous, hypocalcemia, hypokalemia	2 (6)	1 (3)	1 (5)	0 (0)

**Thromboembolic**				
Deep vein thrombosis	0 (0)	5 (14)	0 (0)	4 (18)
Acute myocardial infarction	0 (0)	1 (3)	0 (0)	0 (0)

**Dermatology/Skin**				
Erythemia, rash, desquamation	7 (20)	0 (0)	5 (23)	0 (0)
Alopecia	7 (20)	0 (0)	4 (18)	0 (0)

**Miscellaneous: Other**				
Blurred vision/eye irritation	6 (17)	0 (0)	4 (18)	0 (0)
Mood alteration: agitation, anxiety, depression, hyperactivity	7 (20)	2 (6)	5 (23)	0 (0)

Surgical outcomes are summarized in Table 3. Median blood loss was 500 mL (range 100 – 3000). Median length of stay was 9 d (range 5 – 24 d). Major complications occurred in 6 patients (17%), including delirium (N = 2), liver failure (N = 1), myocardial infarction (N = 1), intraabdominal abscess (N = 1), peripheral neuropathy (N = 1) and death due to an arrhythmia of unknown etiology (N = 1). Thus, operative mortality of liver resection was 3%. The autopsy of the patient who died on the second postoperative day revealed no evidence of thromboembolic (TE) complications or myocardial infarction, but the liver was affected by severe steatohepatitis. Hepatic steatosis was identified in 20 of the 30 pathology reports from surgical specimens (67%).

**Table 3:** Surgical outcomes.

**Surgical Procedures**	
Number of laparotomies	31 (89%)
Number of liver resections	30 (86%)
Number radiofrequency ablations	4 (11%)
**Estimated blood loss (mL)**	728 ± 619
**Days in hospital**	9.6 ± 4.0
**Major complications**	6 (19.4%)
**Mortalities**	1 (3%)

The toxicities related to postoperative chemotherapy are summarized in Table 2. Of the 22 patients who received postoperative chemotherapy, 7 patients (32%) did not have all courses, although 21 patients (96%) had more than 50% of scheduled cycles. Severe adverse events occurred in 5 patients (23%), including DVT (N = 3), severe diarrhea (N = 1) and vomiting (N = 1).

The trial was discontinued early because of a high incidence of TE complications (N = 11; 36%). During preoperative chemotherapy, 6 patients had such complications (including 1 patient with lower extremity DVT and a pulmonary embolus, 4 patients with DVT at the central line (CVL), and 1 patient with myocardial infarction). Two patients were noted perioperatively to have DVTs at the site of the CVL. Three patients had TE complications that first appeared during postoperative chemotherapy, and all of these patients had DVTs at the site of the CVL. In one patient with an upper extremity DVT during preoperative chemotherapy, clot propagation occurred despite being on low molecular weight heparin. While DVT/PE was not considered *a priori *in the stopping rules for the clinical trial, the high incidence and the attendant risks of using anticoagulants at the time of liver surgery was sufficiently compelling to discontinue the trial.

### Survival

**Figure 2:**
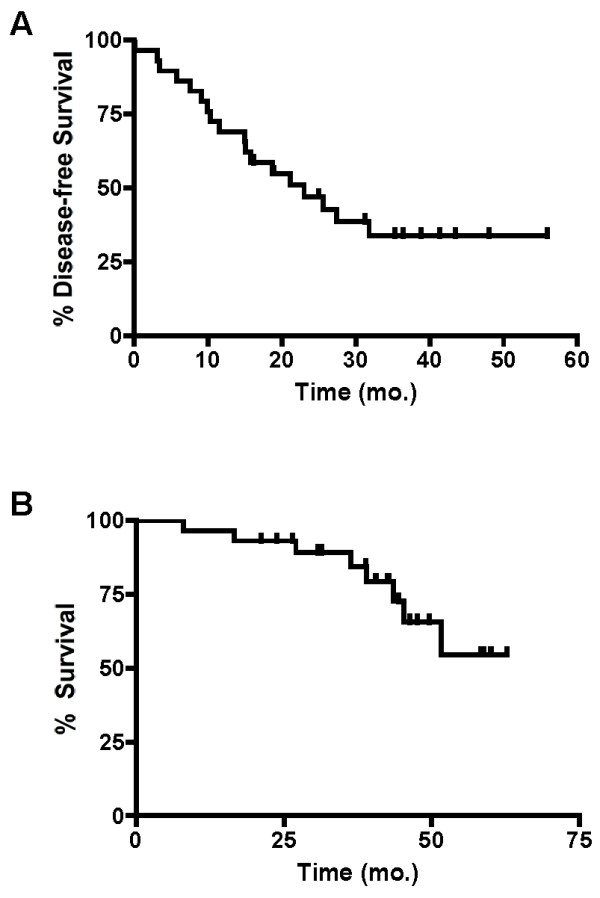
**Kaplan-Meier curves illustrating disease-free survival (A) and overall survival (B) from time of diagnosis, in patients who underwent liver resection**.

Median follow-up for all patients was 38.8 mo. For patients who underwent resection, the median DFS (time of liver resection to time of recurrence) was 23.0 mo (Figure 2A) and 2-year DFS was 47%. Recurrences typically occurred in the liver (N = 2), the lungs (N=4), or both (N = 4). Other sites of recurrence included anastomotic recurrence (N = 2); retroperitoneal lymph nodes (N = 1) and abdominal wall (N = 3). Median disease-free survival was 21.2 and 20.3 mo for patients with a response and patients with stable disease, respectively. Only two patients with progressive disease underwent resection. They recurred at 5.8 and 18.8 mo (median DFS 12.3 mo). The differences between response groups were not statistically significant.

Median OS for patients who underwent resection has not been reached (Figure 2B). Two-year OS for patients who underwent resection was 93%. The two patients with progressive disease who underwent resection survived for 43.5 and 39.0 months without another resection.

**Figure 3:**
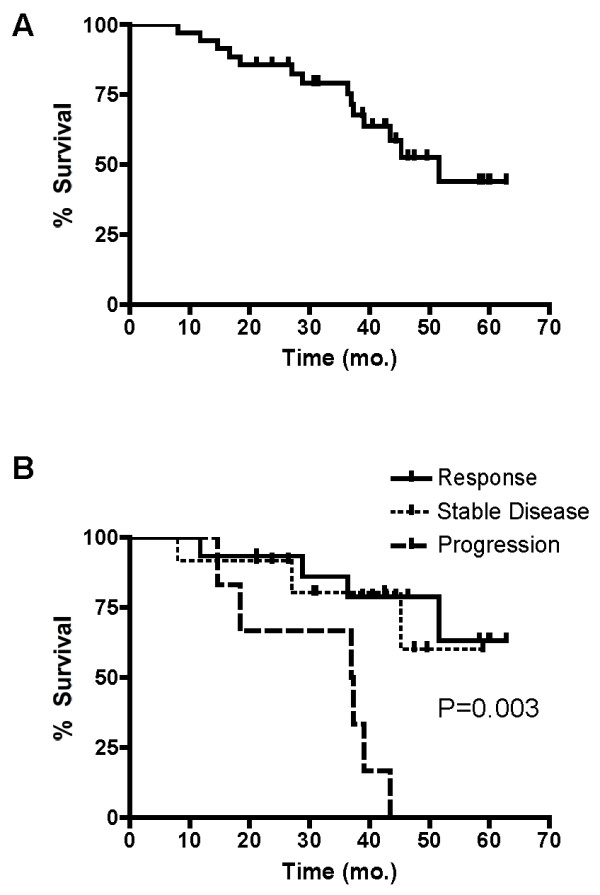
**A Kaplan-Meier curve illustrating overall survival in all patients enrolled in the study**. **B. Overall survivals of all patients enrolled in the study as a function of response to chemotherapy.**

The median OS from the time of diagnosis of CLM (for all patients enrolled in the trial) was 51.6 mo (Figure 3A). If measured from the time of initiation of chemotherapy, median OS for the entire cohort was 51.0 months. At 2 years, OS for the whole cohort was 86%. Overall survival was found to be a function of chemosensitivity. Median OS for those who had a response to chemotherapy and those who had stable disease has not been reached; median OS for those with progressive disease was 37.1 mo from time of diagnosis (and 32.2 mo from the start of chemotherapy). Using an intent-to-treat analysis (which accounts for outcomes related to all patients, including those who did not undergo resection), the relationship between OS and response to chemotherapy was significant (P = 0.003; Figure 3B).

## Discussion

Until recently, most reports on preoperative chemotherapy for CLM focused its utility in the context of unresectable CLM [14-17]. Unresectable CLM can be converted to a resectable state in 3.3 – 32.5% of patients. [12,15,17-19]. In this trial, we have administered preoperative chemotherapy for resectable disease. While the strength of any conclusions on the survival benefits of chemotherapy were diminished by the early cessation of the trial as well as the general design of the trial, it did appear that administration of preoperative CPT-11, 5-FU and LV has utility in selecting patients for surgery and postoperative chemotherapy. However, a number of caveats to this strategy were identified. In particular, there may have been problems related to impaired liver function and there was a high incidence of thromboembolic events.

We have observed a favorable OS (based on an intent-to-treat analysis) of 51.6 months, and 5 liver resections (4 laparotomies) were avoided. Of patients who underwent liver resection, 2-year DFS was 47%. Other reports on the use of chemotherapy for resectable CLM put our results into context. In a series of patients who were selected to receive preoperative FOLFOX and postoperative FOLFIRI or postoperative FOLFOX and FOLFIRI, median time to recurrence was 17 mo, but no long-term outcomes related to preoperative chemotherapy were reported [20]. Resection followed by adjuvant 5-FU and LV was seen to improve DFS over surgery alone in two studies [4,21]. In these trials, OS for patients given postoperative chemotherapy was 47 mo and 62.1 mo, respectively. Thus, we have realized good long-term outcomes in our series despite our intent-to-treat analysis, which takes into account patients who did not undergo resection.

Disease progression was anticipated to be the most concerning adverse outcome. We considered that, if intrahepatic disease progressed to a point where resection was no longer an option, then the preoperative chemotherapy had not served the patient well. This did not occur in any of our patients. In contrast, if an extrahepatic focus of disease appeared while on chemotherapy, then the interval of observation was useful in averting an unnecessary liver resection. This occurred in 3 of our patients (8.6%). Other studies have also demonstrated that neoadjuvant chemotherapy has little impact on technical resectability [22-24].

Most of the liver resections that were avoided were well justified. In addition, only one patient had a laparotomy without resection. This compares favorably to the series reported by Wein et al., who could achieve successful resections in 80% of patients with primarily resectable liver metastases following a course of oxaliplatin-based chemotherapy [25]. This difference may be because, in the latter study, preoperative chemotherapy was administered for much longer (16 – 24 weeks).

The favorable survival seen in those who underwent resection suggests that preoperative chemotherapy provided a means of selecting better candidates for resection. Others have also suggested that preoperative chemotherapy has a selective influence [23,26,27]. In one large retrospective series [26], patients who progressed on preoperative chemotherapy fared poorly after liver resection, with 5-year OS and DFS of only 8% and 3%, respectively. When such a strategy was utilized for patients not judged to have optimally resectable disease, resection was possible in 40% of patients and the authors argued that the lack of response in the remaining was prognostic [27]. The recently reported EORTC trial demonstrated in a randomized controlled fashion an improvement in survival in patients treated with perioperative FOLFOX versus surgery alone [24]. Perioperative chemotherapy was associated with an 8.1% improvement in progression-free survival at 3 years. However, no data were presented that suggested a role for preoperative chemotherapy in the selection of resection candidates. The question remains whether all patients with resectable CLM should receive such a regimen. Until trials comparing neoadjuvant and adjuvant chemotherapy for resectable CLM are done, it may be prudent to administer such a strategy in patients with higher risk of recurrence, such as those with numerous or large CLM; synchronous CLM or short disease-free interval; high serum CEA; equivocal lung lesions on CT scan; or a low likelihood of achieving negative margins [3,5].

Preoperative chemotherapy is not without risk. One potential risk is postoperative liver dysfunction, as chemotherapy is associated with alterations in the hepatic parenchyma such as sinusoidal dilatation, steatosis, and steatohepatitis [28-30]. Steatosis is associated with increased surgical morbidity [30]. Preoperative FOLFOX is associated with a higher risk of postoperative hyperbilirubinemia [24]. Irinotecan-containing regimens are associated with steatohepatitis, and patients with this lesion have an increased surgical mortality [29]. Indeed, we observed that 67% of individuals on this chemotherapy regimen had steatosis in their pathological specimen. One patient had postoperative liver failure. It is possible that the arrhythmic death observed was secondary to impaired hepatic metabolism of drugs. We believe that administration of preoperative chemotherapy (especially those regimens containing irinotecan) is associated with a risk of hepatic dysfunction that might alter outcomes. If neoadjuvant chemotherapy is more widely utilized in the future, some consideration to methods of averting hepatotoxicity should be made.

One risk that was not fully appreciated was the risk of TE complications. Patients had a high baseline risk due to the presence of malignancy and because major surgery was performed. The insertion of indwelling CVLs was an additional risk factor [31]. Significant TE events have previously been reported with CPT-11 and bolus 5-FU/LV [32,33], although such a regimen was only administered to two patients in our study. It is possible that the drug combination itself may be thrombogenic, particularly in the context of surgical patients. The incidence of TE events in our study was higher than anticipated, even compared to another study in which a similar chemotherapy regimen was utilized prior to liver resection [15]. The impact of a TE event during preoperative chemotherapy is considerable, as it necessitates perioperative administration of anticoagulants to patients undergoing surgery associated with a high risk of bleeding. We believe that the high incidence of TE complications could partly be averted by the use of an oral fluoropyrimidine, which would avoid the use of a CVL.

Our experience demonstrates that administration of perioperative chemotherapy is feasible in a setting where patient care can be coordinated through a close collaboration between surgical and medical disciplines. We have demonstrated that the strategy as a whole is associated with good long-term outcomes, even when analyzed in an intent-to-treat fashion. While any conclusions related to survival outcomes have been significantly weakened by the premature cessation of the trial, there is evidence from this trial and from others [23,26,27] that the strategy may be beneficial in selecting the most appropriate patients for resection. Patients who experience clinical deterioration on chemotherapy or who develop extrahepatic disease are removed from the pool of resection candidates. It may also be argued that resection should not be performed at all in patients with progressive disease due to their poor prognosis even with resection. On the other hand, progression in the face of resectable disease may simply spur the use of alternative postoperative systemic treatments, such as bevacizumab or cetuximab. Our data also demonstrate that there are also significant risks to this approach using combined irinotecan, 5-FU and LV. Our findings of a high risk of thromboembolic complications and the association with liver failure have led us to avoid the use of irinotecan preoperatively, particularly over prolonged durations. Future developments should include the avoidance of drugs associated with an increased risk of TE events and use of regimens in which a long-term intravenous access is not necessary. Ultimately, it will be essential to prospectively compare the relative merits of perioperative chemotherapy and postoperative chemotherapy in patients with resectable CLM.

## Conclusion

A short course of chemotherapy prior to hepatic metastasectomy may serve to select candidates best suited for resection and it may also direct postoperative systemic treatment. Given the significant incidence of DVT, alternative systemic neoadjuvant regimens should be investigated, particularly those that avoid the use of a central venous line.

## Abbreviations

The following abbreviations were used: CEA: (carcinoembryonic antigen); CLM: (colorectal liver metastases); CPT-11: (irinotecan); CVL: (central venous line); CR: (complete response); CT: (computer tomography); DFS: (disease-free survival); DVT: (deep venous thrombosis); LV: (leucovorin); MRI: (magnetic resonance imaging); OS: (overall survival); PR: (partial response); SD: (stable disease); RFA: (radiofrequency ablation); TE: (thromboembolic); 5-FU: (5-fluorouracil).

## Competing interests

This study was supported by Pfizer Canada Inc.

Oliver F. Bathe: received honoraria for educational sessions supported by Pfizer Canada Inc ($5,000)

Scott Ernst: received honoraria for educational sessions supported by Pfizer Canada Inc and for participation in advisory boards ($10,000).

All other authors declare that they have no competing interests.

## Authors' contributions

OFB participated in the design of the study, enrolled patients, and drafted the manuscript. SE and SD participated in the design of the study. FRS, ED, CB, DB, DH and GAP enrolled patients on the protocol and made contributions to the methods of protocol implementation. JK coordinated the implementation of the protocol, and collected and analyzed data derived from the study. All authors read and approved the final manuscript.

## Pre-publication history

The pre-publication history for this paper can be accessed here:

http://www.biomedcentral.com/1471-2407/9/156/prepub
